# Distinct Indications for Adjuvant Therapy in Resected Invasive Mucinous Cystic Neoplasms of the Pancreas Compared with Pancreatic Ductal Adenocarcinoma

**DOI:** 10.1245/s10434-024-15841-5

**Published:** 2024-07-27

**Authors:** Paul Wong, Tommaso Pollini, Mohamed A. Adam, Adnan Alseidi, Carlos U. Corvera, Kenzo Hirose, Kimberly S. Kirkwood, Eric K. Nakakura, Lucas Thornblade, Ajay V. Maker

**Affiliations:** Division of Surgical Oncology, Department of Surgery, University of California, San Francisco, CA USA

## Abstract

**Background:**

Surgical and adjuvant management of mucinous cystic neoplasms (MCNs) lacks formal guidelines and data is limited to institutional studies. Factors associated with receipt of adjuvant therapy and any associated impact on survival remain to be clarified. In the absence of other data, guidelines that recommend adjuvant chemotherapy for invasive pancreatic adenocarcinoma have been extrapolated to MCN.

**Patients and Methods:**

The National Cancer Database (2004–2019) was utilized to identify all patients that underwent pancreatic resection for invasive MCNs. Patients that received neoadjuvant therapy or did not undergo lymphadenectomy were excluded. Patient, tumor, and treatment factors associated with survival were assessed.

**Results:**

For 161 patients with invasive MCN, median overall survival (OS) was 133 months and 45% of patients received adjuvant therapy. Multivariable analysis demonstrated that poorly differentiated tumors [odds ratio (OR) 4.19, 95% confidence interval (CI) 1.47–11.98; *p *= 0.008] and positive lymph node status (OR 2.67, 95% CI 1.02–6.98; *p *= 0.042) were independent predictors of receiving adjuvant therapy. Lymph node positivity [hazard ratio (HR) 2.90, 95% CI 1.47–5.73; *p *= 0.002], positive margins (HR 5.28, 95% CI 2.28–12.27; *p *< 0.001), and stage III disease (HR 12.46, 95% CI 1.40–111.05; *p *= 0.024) were associated with worse OS. Receipt of adjuvant systemic therapy was independently associated with decreased risk of mortality in node positive patients (HR 0.23, 95% CI 0.10–0.69; *p *= 0.002). Survival was not associated with adjuvant therapy in patients with negative lymph nodes or margin negative status.

**Conclusion:**

In contrast to pancreatic ductal adenocarcinoma (PDAC), where adjuvant therapy improves OS for every tumor stage, surgery alone for invasive MCN is not associated with improved OS compared with surgery plus adjuvant therapy in node-negative patients. Surgery alone is likely sufficient for a subset of invasive MCN.

Mucinous cystic neoplasms represent uncommon pancreatic tumors characterized by ovarian-type stroma beneath a layer of mucin-secreting cells. These tumors are predominantly observed in females aged between 40 and 60 years old.^1^ While most MCNs are benign, these tumors can progress to adenocarcinoma.^1^ Similar to intraductal papillary mucinous neoplasms (IPMNs), both tumors are categorized as mucinous, displaying varying degrees of dysplasia in their epithelium, ranging from low to high grade. Some lesions may advance to include an associated invasive component.

The International Association of Pancreatology recommends surgical removal of all MCNs regardless of size, while the European guidelines endorse a surveillance approach for MCNs < 40 mm if asymptomatic and without high-risk features (i.e., mural nodules).^2,3^ While management guidelines have incorporated the consideration of surgical resection for MCNs, the question of adjuvant treatment has not been comprehensively addressed. There is a paucity of studies assessing the use of adjuvant therapy for invasive MCNs due to the inadequate sample sizes of these lesions in institutional studies. The European guidelines recommended adjuvant treatment for invasive MCN based on an extrapolation from sporadic pancreatic adenocarcinomas. The decision was made due to the cited lack of evidence either supporting or refuting this approach. This absence of centralized adjuvant treatment guidelines for MCNs has left the question of treatment administration up to multidisciplinary teams at referral centers or individual oncologists.

Thus, there remains a need to validate the utility of adjuvant therapy for invasive MCNs and to determine the specific subsets of patients that may benefit from systemic therapy following resection. In this study, a nationally validated outcomes database was used to assess whether adjuvant systemic therapy is associated with survival in patients that underwent resection for invasive mucinous cystic neoplasms.

## Patients and Methods

### Patient Population

Following approval from the institutional review board (IRB), data from the National Cancer Database (NCDB) were used. Established in 1989, the NCDB is a joint project between the American Cancer Society and the Commission on Cancer (CoC) of the American College of Surgeons. The database captures approximately 72% of all newly diagnosed malignancies in the USA and incorporates comprehensive clinical oncology data from CoC-accredited facilities.^4^ The Participant Use Data Files contain deidentified and Health Insurance Portability and Accountability Act (HIPAA)-compliant data for investigators of CoC-accredited institutions.

**Fig. 1:**
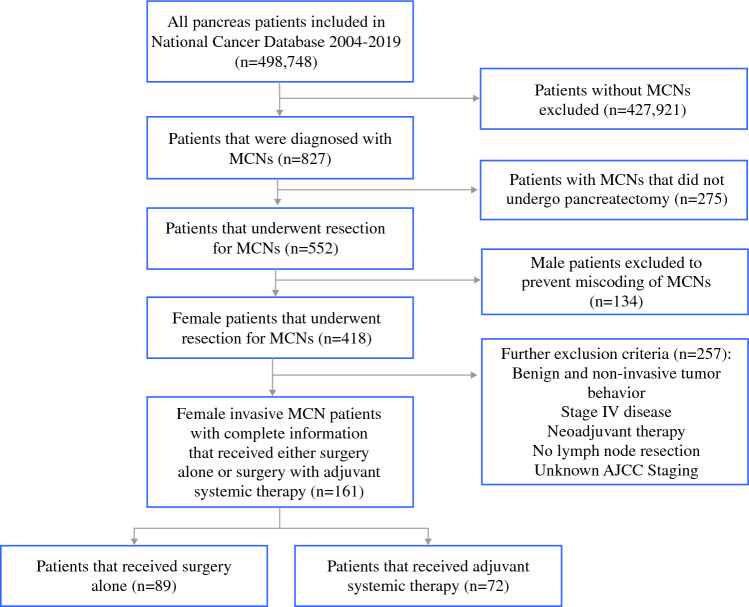
Flow diagram of selection criteria for all patients with invasive mucinous cystic neoplasms (MCNs) that either received surgery alone or surgery with adjuvant systemic therapy

Patients with invasive mucinous cystic neoplasms (MCNs) that received surgical resection from 2004–2019 were identified through the NCDB (Fig. 1). Patients were excluded if they received neoadjuvant therapy, did not undergo lymphadenectomy, or had unknown American Joint Committee on Cancer (AJCC) staging information. Male patients were excluded to address potential miscoding of MCNs. The cohort of patients was then subdivided into those that received surgery alone and those that received surgical resection followed by adjuvant systemic therapy. Potential clinically relevant demographic and clinicopathologic confounding variables (Table 1) were adjusted for in logistic regression models.

**Table 1:** Predictors of receiving adjuvant systemic therapy versus surgery alone in patients with invasive mucinous cystic neoplasms

Variable	Univariable (*n*, %)				Multivariable	
	All patients (*n* = 161)	Surgery alone (*n* = 89)	Adjuvant systemic therapy (*n* = 72)	*p* value	OR [95% CI]	*p* value
Age						
< 50 years	50 (31.1)	31 (34.8)	19 (26.4)	0.250		
Race/ethnicity						
Caucasian	131 (81.4)	74 (83.1)	57 (79.2)	0.937		
Black	20 (12.4)	10 (11.2)	10 (13.9)			
Asian	4 (2.5)	2 (2.2)	2 (2.8)			
Other	6 (3.7)	3 (3.4)	3 (4.2)			
Patient county population						
Metro	128 (79.5)	64 (71.9)	64 (88.9)	**0.020**	**Ref.**	**–**
Urban	22 (13.7)	16 (18.0)	6 (8.3)		0.39 [0.14–1.08]	0.07
Rural	4 (2.5)	2 (2.2)	2 (2.8)		0.89 [0.10–8.11]	0.917
Unknown	7 (4.3)	7 (7.9)	0 (0.0)			
Facility type						
Academic	73 (45.3)	44 (49.4)	29 (40.3)	0.051		
Nonacademic	67 (41.6)	30 (33.7)	37 (51.4)			
Unknown	21 (13.0)	15 (16.9)	6 (8.3)			
Insurance status						
Private	80 (49.7)	42 (47.2)	38 (52.8)	0.759		
Nonprivate	79 (49.1)	46 (51.7)	33 (45.8)			
Unknown	2 (1.2)	1 (1.1)	1 (1.4)			
Charlson–Deyo score						
0	103 (64.0)	57 (64.0)	46 (63.9)	0.568		
1	42 (26.1)	21 (23.6)	21 (29.2)			
2	11 (6.8)	7 (7.9)	4 (5.6)			
3+	5 (3.1)	4 (4.5)	1 (1.4)			
Tumor size						
< 5 cm	73 (45.3)	40 (44.9)	33 (45.8)	0.974		
≥ 5 cm	83 (51.6)	46 (51.7)	37 (51.4)			
Unknown	5 (3.1)	3 (3.4)	2 (2.8)			
Tumor site category						
Head	33 (20.5)	22 (24.7)	11 (15.3)	0.301		
Body/tail	107 (66.5)	57 (64.0)	50 (69.4)			
Other or NOS	21 (13.0)	10 (11.2)	11 (15.3)			
Pancreatectomy type						
Pancreaticoduodenectomy	59 (36.6)	33 (37.1)	26 (36.1)	0.661		
Partial pancreatectomy	81 (50.3)	42 (47.2)	39 (54.2)			
Total pancreatectomy	14 (8.7)	9 (10.1)	5 (6.9)			
Other, NOS	7 (4.3)	5 (5.6)	2 (2.8)			
Grade/differentiation						
Well/moderately differentiated	89 (55.3)	54 (60.7)	35 (48.6)	**0.020**	**Ref.**	**–**
Poorly differentiated/anaplastic	24 (14.9)	7 (7.9)	17 (23.6)		4.19 [1.47–11.98]	**0.008**
Indeterminate	48 (29.8)	28 (31.5)	20 (27.8)		1.14 [0.54–2.40]	0.740
TNM stage						
I	101 (62.7)	61 (68.5)	40 (55.6)	0.152		
II	59 (36.6)	28 (31.5)	31 (43.1)			
III	1 (0.6)	0 (0.0)	1 (1.4)			
Lymph nodes examined						
1–14 nodes	107 (66.5)	55 (61.8)	52 (72.2)	0.164		
15+ nodes	54 (33.5)	34 (38.2)	20 (27.8)			
Lymph node positivity						
Node negative	137 (85.1)	80 (89.9)	57 (79.2)	**0.047**	**Ref**	**–**
Node positive	24 (14.9)	9 (10.1)	15 (20.8)		2.67 [1.02–6.98]	**0.042**
Resection margin						
R0	147 (91.3)	83 (93.3)	64 (88.9)	0.585		
R1/R2	10 (6.2)	4 (4.5)	6 (8.3)			
Unknown	4 (2.5)	2 (2.2)	2 (2.8)			

Bold values indicate *p* < 0.05

### Statistical Analysis

Categorical variables were presented as proportions, and statistical associations were calculated using the chi-squared test. Binary logistic regression models were utilized to determine the likelihood of receiving adjuvant therapy and were reported as odds ratio (OR) with 95% confidence interval (CI). Univariable estimates of overall survival were calculated using the Kaplan–Meier method, and comparisons between groups were conducted using the log-rank test. Multivariable Cox proportional hazard models were constructed using backward stepwise variable selection to compare overall survival (OS) among patients with invasive MCN. This included all variables that were identified as significantly associated with improved survival in univariable analysis. Two tailed *p* values < 0.05 were used as the threshold for statistical significance. All statistical analyses were performed using the SPSS 27 software (IBM, Armonk, NY).

## Results

### Patient Characteristics and Use of Adjuvant Therapy

Of the 161 patients with invasive MCN that met all study criteria, the median age was 60 years (IQR, 46–71 years). The majority of patients were Caucasian without comorbidities (Charlson–Deyo score 0) and resided in a metropolitan area (Table 1). In addition, 45.3% (*n* = 73) of patients were treated at an academic hospital and 49.7% (*n* = 80) had private insurance. Most patients had a tumor ≥ 5 cm, location in the body or tail, underwent partial pancreatectomy, and had a negative resection margin (R0) (Table 1). Lymphadenectomy of ≥ 15 lymph nodes was performed in 33.5% (*n* = 54) of patients, with lymph node positivity (N1) in 14.9% (*n* = 24) of patients. A total of 62.7% (*n* = 101) of patients were stage I, 36.6% (*n* = 59) were stage II, and 0.6% (*n* = 1) of patients had stage III disease.

In the overall cohort, 72 (44.7%) patients received adjuvant systemic therapy whereas 89 (55.3%) underwent surgery alone. Multivariable regression analysis was performed to highlight the clinicopathologic and patient characteristics that predicted adjuvant therapy receipt. Poorly differentiated/anaplastic tumor grade (OR 4.19, 95% CI 1.47–11.98; *p* = 0.008) and node positivity (OR 2.67, 95% CI 1.02–6.98; *p* = 0.042) were found to be independent predictors of adjuvant therapy receipt (Table 1).

### Survival Analysis

**Fig. 2:**
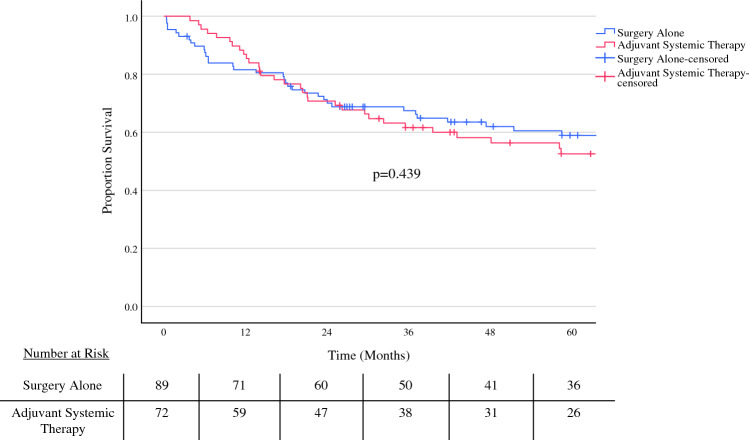
Kaplan–Meier curve depicting overall survival of patients with invasive mucinous cystic neoplasm (MCN) that either received adjuvant systemic therapy or surgery alone

For the entire cohort, the median overall survival (OS) for patients with invasive MCN was 133.2 months, with 3- and 5-year survival rates of 65% and 56%, respectively (Fig. 2). Kaplan–Meier survival analysis comparing adjuvant therapy versus surgery alone showed no difference in median overall survival for the entire cohort (*p* = 0.44). To assess which clinicopathologic and operative characteristics were predictors of mortality, Cox proportional hazard models were generated (Table 2). After adjusting for potential clinically relevant confounders, positive lymph node status [hazard ratio (HR) 2.90, 95% CI 1.47–5.73; *p* = 0.002], positive margins (HR 5.28, 95% CI 2.28–12.27; *p* < 0.001), and AJCC TNM stage III disease (HR 12.46, 95% CI 1.40–111.05; *p* = 0.024) were associated with worse OS. On the other hand, pancreatic body/tail tumor location was an independent predictor of improved OS compared with tumors in the head (HR 0.56, 95% CI 0.32–0.97; *p* = 0.037).

**Table 2:** Cox logistic regression models identifying predictors of overall survival in patients with invasive mucinous cystic neoplasms

Variable	Univariable		Multivariable	
	HR [95% CI]	*p* value	HR [95% CI]	*p* value
Age				
50+ years	2.15 [1.20–3.85]	**0.009**	1.48 [0.76–2.87]	0.249
Race/ethnicity				
Caucasian	**Ref.**	**–**		
Black	0.57 [0.25–1.32]	0.263		
Asian	1.56 [0.49–4.97]	0.455		
Other	0.923 [0.23–3.78]	0.911		
Patient county population				
Metro	**Ref.**	**–**		
Urban	0.77 [0.37–1.61]	0.481		
Rural	0.82 [0.20–3.36]	0.781		
Facility type				
Academic	**Ref.**	**–**		
Non-Academic	1.50 [0.93–2.43]	0.098		
Insurance status				
Private	**Ref.**	**–**		
Nonprivate	1.10 [0.69–1.75]	0.688		
Charlson–Deyo score				
0	**Ref.**	**–**		
1	1.09 [0.64–1.87]	0.742		
2	1.13 [0.45–2.86]	0.791		
3+	1.31 [0.41–4.23]	0.648		
Tumor size				
< 5 cm	**Ref.**	**–**		
≥ 5 cm	1.11 [0.69–1.78]	0.666		
Tumor site category				
Head	**Ref.**	**–**	**Ref.**	**–**
Body/tail	0.54 [0.32–0.91]	**0.021**	0.56 [0.32–0.97]	**0.037**
Other or NOS	0.46 [0.20–1.04]	0.062	0.33 [0.14–0.77]	**0.011**
Pancreatectomy type				
Pancreaticoduodenectomy	**Ref.**	**–**		
Partial pancreatectomy	0.62 [0.38–1.02]	0.062		
Total pancreatectomy	0.92 [0.41–2.11]	0.849		
Other, NOS	0.37 [0.09–1.55]	0.172		
Grade/differentiation				
Well/moderately differentiated	**Ref.**	**–**		
Poorly differentiated/anaplastic	0.63 [0.30–1.32]	0.221		
Indeterminate	0.42 [0.23–0.78]	**0.006**		
TNM stage				
I	**Ref.**	**–**	**Ref.**	**–**
II	2.42 [1.51–3.87]	**< 0.001**	1.32 [0.72–2.42]	0.365
III	22.85 [2.87–181.71]	**0.003**	12.46 [1.40–111.05]	**0.024**
Lymph nodes examined				
1–15 nodes	**Ref.**	**–**		
15+ nodes	0.79 [0.47–1.33]	0.382		
Lymph node positivity				
Node negative	**Ref.**	**–**	**Ref.**	**–**
Node positive	4.13 [2.48–6.88]	**< 0.001**	2.90 [1.47-5.73]	**0.002**
Resection margin				
R0	**Ref.**	**–**	**Ref**	**–**
R1/R2	8.94 [4.17–19.17]	**< 0.001**	5.28 [2.28-12.27]	**< 0.001**
Postoperative treatment				
Surgery alone	**Ref.**	**–**		
Adjuvant systemic therapy	1.20 [0.76–1.91]	0.440		

Bold values indicate *p* < 0.05

In patients with invasive MCN that received adjuvant therapy, Kaplan–Meier estimates demonstrated that TNM stages II–III, lymph node positivity, and positive resection margin status were associated with a worse OS (all *p* < 0.05) (Table 3). Following multivariable analysis, TNM stage III and positive resection margin status remained independent predictors of worse OS in patients that received adjuvant therapy (both *p* < 0.001).

**Table 3:** Predictors of overall survival in patients with invasive mucinous cystic neoplasms that received adjuvant systemic therapy

Variable	Univariable		Multivariable	
	HR [95% CI]	*p* value	HR [95% CI]	*p* value
Age				
50+ years	2.75 [1.06–7.11]	**0.037**	1.70 [0.62–4.60]	0.301
Race/ethnicity				
Caucasian	**Ref.**	**–**		
Black	0.53 [0.16–1.73]	0.289		
Asian	1.49 [0.20–11.10]	0.698		
Other	1.47 [0.20–10.91]	0.706		
Patient county population				
Metro	**Ref.**	**–**		
Urban	1.17 [0.36–3.82]	0.800		
Rural	2.38 [0.56–10.07]	0.239		
Facility type				
Academic	**Ref.**	**–**		
Nonacademic	1.64 [0.82–3.28]	0.161		
**Insurance status**				
Private	**Ref.**	**–**		
Nonprivate	1.04 [0.53–2.04]	0.908		
Charlson–Deyo score				
0	**Ref.**	**–**		
1	0.73 [0.33–1.62]	0.435		
2	1.80 [0.54–6.05]	0.341		
3+	6.10 [0.77–48.38]	0.087		
Tumor size				
< 5 cm	**Ref.**	**–**		
≥ 5 cm	0.96 [0.53–1.73]	0.886		
Tumor site category				
Head	**Ref.**	**–**		
Body/tail	0.50 [0.21–1.19]	0.117		
Other or NOS	0.48 [0.15–1.52]	0.212		
Pancreatectomy type				
Pancreaticoduodenectomy	**Ref.**	**–**		
Partial pancreatectomy	0.66 [0.32–1.37]	0.263		
Total pancreatectomy	1.33 [0.38–4.69]	0.654		
Other, NOS	2.30 [0.51–10.26]	0.277		
Grade/differentiation				
Well/moderately differentiated	**Ref.**	**–**		
Poorly differentiated/anaplastic	0.69 [0.28–1.69]	0.414		
Indeterminate	0.50 [0.21–1.17]	0.110		
TNM stage				
I	**Ref.**	**–**	**Ref.**	**–**
II	1.98 [1.01–3.91]	**0.048**	1.67 [0.82–3.42]	0.159
III	95.97 [5.83–1580.55]	**0.001**	135.49 [7.97–2302.28]	**< 0.001**
Lymph nodes examined				
1–15 nodes	**Ref.**	**–**		
15+ nodes	0.53 [0.22–1.29]	0.162		
Lymph node positivity				
Node negative	**Ref.**	**–**	**Ref.**	**–**
Node positive	3.18 [1.59–6.36]	**0.001**	2.01 [0.78–5.20]	0.151
Resection margin				
R0	**Ref.**	**–**	**Ref.**	**–**
R1/R2	7.32 [2.58–20.75]	**< 0.001**	6.47 [2.18–19.24]	**< 0.001**

Bold values indicate *p* < 0.05

**Fig. 3:**
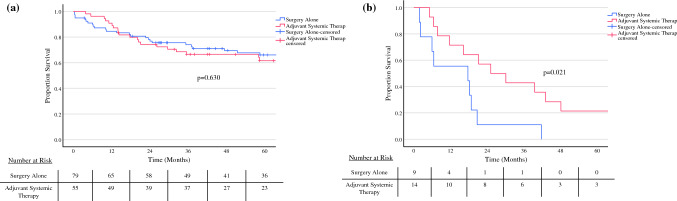
Kaplan–Meier curve demonstrating overall survival of patients with invasive mucinous cystic neoplasm (MCN) with (**a**) node-negative and (**b**) node-positive disease receiving adjuvant systemic therapy or surgery alone

A subanalysis was performed that included only patients with invasive MCN with positive lymph node status (N1) (Table 4). Univariable analysis demonstrated that tumor size ≥ 5cm, partial pancreatectomy, body/tail tumor location, and receiving adjuvant systemic therapy were associated with improved OS, while positive resection margin status corresponded with worse OS (all *p* < 0.05). On multivariable analysis, partial pancreatectomy was significantly associated with better OS (HR 0.16, 95% CI 0.05–0.53; *p* = 0.002), and receipt of adjuvant therapy was an independent predictor of decreased mortality (HR 0.23, 95% CI 0.10–0.69; *p* = 0.002). Notably, in node negative (N0) patients, survival was not associated with the addition of adjuvant therapy (Fig. 3).

**Table 4:** Predictors of overall survival in patients with invasive mucinous cystic neoplasms and positive lymph node status (N1)

Variable	Univariable		Multivariable	
	HR [95% CI]	*p* value	HR [95% CI]	*p* value
Age				
50+ years	0.047 [0.003–0.74]	**0.030**	0.15 [0.01–2.48]	0.184
Race/ethnicity				
Caucasian	**Ref.**	**–**		
Black	2.02 [0.25–16.28]	0.507		
Asian	0.92 [0.12–7.08]	0.937		
Other	2.74 [0.33–22.67]	0.349		
Patient county population				
Metro	**Ref.**	**-**		
Urban	2.62 [0.73–9.50]	0.141		
Rural	0.53 [0.07–4.12]	0.548		
Facility type				
Academic	**Ref.**	**-**		
Nonacademic	0.51 [0.20–1.32]	0.168		
Insurance status				
Private	**Ref.**	**–**		
Nonprivate	0.94 [0.38–2.29]	0.887		
Charlson–Deyo score				
0	**Ref.**	**–**		
1	2.23 [0.84–5.92]	0.108		
2	2.84 [0.56–14.50]	0.208		
3+	–	–		
Tumor size				
< 5 cm	**Ref.**	**–**		
≥ 5 cm	0.58 [0.24–1.38]	0.216		
Tumor site category				
Head	**Ref**	**–**	**Ref.**	**–**
Body/tail	0.31 [0.11–0.90]	**0.031**	0.45 [0.04–5.48]	0.534
Other or NOS	0.28 [0.08–1.05]	0.060	0.43 [0.04–4.36]	0.478
Pancreatectomy type				
Pancreaticoduodenectomy	**Ref.**	**–**	**Ref.**	**–**
Partial pancreatectomy	0.21 [0.07–0.62]	**0.005**	0.16 [0.05–0.53]	**0.002**
Total pancreatectomy	1.01 [0.12–8.28]	0.995	0.44 [0.05–3.80]	0.455
Other, NOS	1.42 [0.17–11.92]	0.749	2.81 [0.30–26.38]	0.365
Grade/differentiation				
Well/moderately differentiated	**Ref.**	**–**		
Poorly differentiated/anaplastic	0.96 [0.27–3.40]	0.947		
Indeterminate	1.02 [0.38–2.69]	0.975		
TNM stage				
I	**Ref.**	**–**		
II	0.65 [0.08–5.10]	0.683		
III	7.00 [0.33–150.76]	0.214		
Lymph nodes examined				
1–15 nodes	**Ref.**	**–**		
15+ nodes	1.93 [0.72–5.20]	0.192		
Resection margin				
R0	**Ref.**	**–**	**Ref.**	**–**
R1/R2	3.45 [1.14-10.44]	**0.028**	2.00 [0.49–8.12]	0.332
Postoperative treatment				
Surgery alone	**Ref.**	**–**	**Ref.**	**–**
Adjuvant systemic therapy	0.34 [0.13–0.88]	**0.026**	0.23 [0.10–0.69]	**0.002**

Bold values indicate *p* < 0.05

For comparison, patients with pancreatic ductal adenocarcinoma (PDAC) over the same time frame were evaluated. Adjuvant therapy for PDAC was associated with improved OS (median OS: surgery alone, 13.5 months vs. adjuvant chemotherapy, 24.0 months; *p* < 0.001). The associated improvement in OS persisted for node negative PDAC (median OS: surgery alone, 22.2 months vs. adjuvant chemotherapy, 34.4 months; *p* < 0.001), and node positive PDAC (median OS: surgery alone, 11.1 months vs. adjuvant chemotherapy, 21.4 months; *p* < 0.001).

## Discussion

The role of adjuvant therapy in the treatment of invasive mucinous cystic neoplasms of the pancreas was investigated using a nationally validated outcomes database. To our knowledge, this is the largest study that has assessed the efficacy of adjuvant therapy and the clinicopathologic variables associated with its administration in a heterogeneous cohort of patients with MCN. It was shown that poorly differentiated/anaplastic tumor grade and node positivity were independent predictors of adjuvant therapy receipt. Notably, other variables such as age, tumor size (≥ 5 cm), hospital type, tumor site, and AJCC stage were not found to be significantly associated with the administration of adjuvant therapy (*p* > 0.05). Positive lymph node status, positive margins, and AJCC stage III disease were independent predictors of worse overall survival, whereas pancreatic body/tail tumor location was a significant predictor of decreased mortality. Interestingly, in the entire cohort, there was no difference in median overall survival between patients that underwent surgery alone or received adjuvant therapy. However, when considering only node-positive (N1) patients, omission of adjuvant chemotherapy was an independent predictor of increased mortality.

The mainstay of curative treatment for MCNs remains surgical resection of the tumor, with careful pathologic examination for the presence of an invasive component in the lesion. Previous surgical series have reported that as many as 34% of MCNs contain invasive cancer, and that MCNs larger than 4 cm are associated with a higher risk of malignant transformation.^5,6^ However, more recent series and a systematic review suggest that the incidence of malignancy is likely considerably lower than previously reported, and that a larger size cutoff for malignancy risk may be more reasonable.^7^ Invasive MCNs carry a worse prognosis compared with noninvasive MCNs; however, their outcomes are still better than that of sporadic PDAC.^1^

This study is significant as it addresses the lack of clear evidence or guidelines to guide the administration of systemic adjuvant treatment for invasive mucinous tumors. Unlike the classic PanIn to tubular PDAC sequence, the underlying histology and progression pathways of these tumors are distinct.^8,9^ The findings provide valuable insights for guiding the post-resection management of patients with MCN, particularly considering the distinct nature of their histology and progression.

**Fig. 4:**
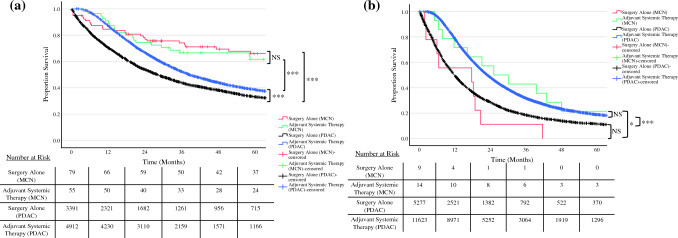
Kaplan–Meier curve demonstrating overall survival of patients with invasive mucinous cystic neoplasm (MCN) and pancreatic ductal adenocarcinoma (PDAC) with (**a**) node-negative and (**b**) node-positive disease receiving adjuvant systemic therapy or surgery alone. NS, *p* > 0.05, *p < 0.05, **p < 0.01, ***p < 0.001. *NS* not significant

The European Study Group on Cystic Tumours of the Pancreas guidelines suggest an equivalence in adjuvant treatment for MCNs and sporadic pancreatic adenocarcinoma but note insufficient evidence at the time of their statement.^3^ It is important to note that previous trials indicate a survival benefit for both node-negative and node-positive patients with PDAC with adjuvant chemotherapy, and we confirmed these findings in the current data set^10,11^ (Fig. 4). However, this study demonstrates that node-negative patients with MCN may not derive a survival advantage with adjuvant therapy, unlike their node-positive counterparts. Overall survival of invasive node-negative MCN is significantly improved compared with node-negative PDAC; however, it is comparable in the node positive setting. Adjuvant therapy after PDAC resection typically follows international and NCCN guidelines, involving either modified FOLFIRINOX or a gemcitabine-based regimen.^12–14^ Side effects, including fatigue, nausea, vomiting, and peripheral neuropathy, are common, necessitating a need to minimize systemic treatment to prevent overtreatment, especially for PDACs measuring < 1 cm, where adjuvant therapy may not confer a survival advantage.^15^ Thus, there is a need to minimize the amount of systemic treatment administered to patients.^16^

The management of pancreatic cystic neoplasms, particularly in IPMNs and MCNs, offers opportunities to reduce overtreatment. Despite revised treatment guidelines, unnecessary surgery occurs for low-grade dysplasia lesions. In fact, Khoury et al. demonstrated that only 23% of resected IPMNs in the USA contained invasive or high-grade histology.^17^ Likewise, for MCNs, a recent systematic review and meta-analysis found the rate of malignancy in these lesions to be roughly 16%, which provides an argument that surveillance is sufficient for most patients despite current guidelines recommending resection of all MCNs.^7^ This trend against overtreatment may extend to adjuvant therapy, focusing on specific subsets of patients with MCN that may benefit from systemic treatment, considering the relative rarity of invasive MCNs.

There were several limitations in this study. First, while the National Cancer Database poses a unique advantage by encompassing a large sample of patients, the database does not contain granular variables regarding the specific chemotherapy regimens prescribed in the adjuvant setting, the duration of treatment, and the associated toxicities of adjuvant treatment. This exclusion of exact treatment data prevents further analyses comparing surgery alone with various durations and types of adjuvant treatment, along with potential toxicities that patients developed with systemic therapy. We did analyze a subset of patients from a more recent timeframe, spanning from 2010 to 2019. This period included a more recent cohort and was chosen because it also represents the period after the reporting of the Groupe Tumeurs Digestives of Unicancer and the PRODIGE Intergroup randomized controlled trial demonstrating the superiority of FOLFIRINOX over gemcitabine for metastatic pancreatic cancer. The results of this subanalysis, namely of an association of adjuvant chemotherapy with survival only in the node-positive cohort, were not different than the results that included all patients in the time period. Further, the NCDB lacks recurrence and disease-specific mortality data, limiting the ability to evaluate recurrence-free and disease-specific survival. With regards to the patients with invasive MCN that were not operated on, the NCDB does not have information on indications of surgery or lack thereof. In addition, other limitations that are characteristic of retrospective databases were present in this study, which included missing data and errors in adjuvant treatment and histologic classification. The cases were selected based on the International Classification of Diseases (ICD)-O-3 code for MCN only, and the codes for IPMN were excluded; nonetheless, the possibility of misclassification bias exists. MCNs were defined and further differentiated from IPMNs by the presence of ovarian stroma in the cyst lining based on the World Health Organization 2000 classification, and our cohort was thus chosen to start 4 years after this definition in 2004. Regardless, the diagnostic criteria of MCN have become increasingly standardized over time, and thus we excluded male patients from the analysis to prevent including those that were potentially miscoded/misdiagnosed, especially early in the series. Specifically, an initial review of the biological sex distribution of resected MCNs yielded 24% males. It should be noted that the eight-institution Central Pancreas Consortium evaluated their 14-year experience with MCN and found the incidence of MCN was similar and up to 15% in men.^1^ Regardless, as MCNs occur most commonly in biological females, the limitations of histologic coding in the database were considered and ultimately prompted the exclusion of male patients from this study to prevent potential miscoding of these lesions.^5,19^

## Conclusions

In summary, our study indicates that patients with invasive mucinous cystic neoplasms (MCNs) and node-positive disease experience improved overall survival associated with adjuvant systemic therapy in comparison to those undergoing surgery alone. Notably, this association is not evident in patients with node-negative disease. This finding stands in contrast to pancreatic ductal adenocarcinomas, where both node-negative and node-positive patients demonstrate improved survival with adjuvant systemic therapy. This supports the assertion that there may be distinct indications for adjuvant therapy in resected invasive mucinous cystic neoplasms of the pancreas compared with pancreatic ductal adenocarcinoma.
